# LIPSS-based functional surfaces produced by multi-beam nanostructuring with 2601 beams and real-time thermal processes measurement

**DOI:** 10.1038/s41598-021-02290-3

**Published:** 2021-11-25

**Authors:** P. Hauschwitz, J. Martan, R. Bičišťová, C. Beltrami, D. Moskal, A. Brodsky, N. Kaplan, J. Mužík, D. Štepánková, J. Brajer, D. Rostohar, J. Kopeček, L. Prokešová, M. Honner, V. Lang, M. Smrž, T. Mocek

**Affiliations:** 1 Hilase Centre, Institute of Physics, Academy of Sciences of the Czech Republic, Za Radnici 828, Dolni Brezany, 25241 Czech Republic; 2 New Technologies Research Centre (NTC), University of West Bohemia, Univerzitni 8, 30100 Plzen, Czech Republic; 3 R&D Department, Holo/Or Ltd, Einstein 13b, 7403617 Ness Tziona, Israel; 4 Faculty of Nuclear Sciences and Physical Engineering, Czech Technical University in Prague, Brehova 7, 115 19 Prague, Czech Republic; 5 Institute of Physics of the Czech Academy of Sciences, Na Slovance 2, Prague, Czech Republic

**Keywords:** Lasers, LEDs and light sources, Ultrafast lasers

## Abstract

A unique combination of the ultrashort high-energy pulsed laser system with exceptional beam quality and a novel Diffractive Optical Element (DOE) enables simultaneous production of 2601 spots organized in the square-shaped 1 × 1 mm matrix in less than 0.01 ms. By adjusting the laser and processing parameters each spot can contain Laser Induced Periodic Surface Structures (LIPSS, ripples), including high-spatial frequency LIPSS (HFSL) and low-spatial frequency LIPSS (LSFL). DOE placed before galvanometric scanner allows easy integration and stitching of the pattern over larger areas. In addition, the LIPSS formation was monitored for the first time using fast infrared radiometry for verification of real-time quality control possibilities. During the LIPSS fabrication, solidification plateaus were observed after each laser pulse, which enables process control by monitoring heat accumulation or plateau length using a new signal derivation approach. Analysis of solidification plateaus after each laser pulse enabled dynamic calibration of the measurement. Heat accumulation temperatures from 200 to 1000 °C were observed from measurement and compared to the theoretical model. The temperature measurements revealed interesting changes in the physics of the laser ablation process. Moreover, the highest throughput on the area of 40 × 40 mm reached 1910 cm^2^/min, which is the highest demonstrated throughput of LIPSS nanostructuring, to the best of our knowledge. Thus, showing great potential for the efficient production of LIPSS-based functional surfaces which can be used to improve surface mechanical, biological or optical properties.

## Introduction

Surface micro and nanostructuring is a hot topic in many research facilities around the world. Suitable shaped surface features can modify surface properties to attain anti-icing^1^, anti-corrosion, superhydrophobicity and self-cleaning^2,3^, control of friction^4^, anti-reflection^5,6^, anti-bacteria^7^ properties or serve as a decoration^8^.

Conventional nanostructuring techniques including lithography^9^, sol-gel^10^, thermal embossing^11^, plasma treatments^12^, chemical vapor deposition^13^, chemical etching^14^ or electrodeposition^15^ require chemicals or long fabrication time due to slow or complicated multi-step processes. The possible alternative is direct laser ablation as a single-step, non-polluting, cost-effective and flexible method for efficient and high-precision processing^16–18^.

Laser-Induced Periodic Surface Structures (LIPSS) presents a simple and robust way for surface nanostructuring. By utilizing ultrashort pulsed laser systems, LIPSS can be reliably generated in a single step process and without the use of expensive and slow vacuum technology and lithography^19^. LIPSS can be classified by their period to Low Spatial Frequency LIPSS (LSFL) with the periodicity close to the laser wavelength. LSFL can be observed mostly on metals and semiconductors with orientation generally dependent on the laser beam polarization and laser processing conditions^20^. The second common type are High Spatial Frequency LIPSS (HSFL) characterized by the period smaller than half of the laser wavelength. Depending on the material, the orientation of HSFL can be parallel or perpendicular to the beam polarization^21^. Except the wavelength, the periodicity of LIPSS can be controlled by the angle of incidence, fluence or by the applied number of pulses, while the direction of LIPSS can be controlled by the polarization of laser beam^22^. The great flexibility and variability of produced LIPSS and ripple structures following the laser and processing parameters allows tailoring surface mechanical, biological or optical properties^19,22^.

However, the wider industrial use of LIPSS-based functional surfaces is limited by processing speed. Generally, throughputs only up a few cm^2^ per minute are reached by conventional single beam approaches^19^. In addition, speeding up the single beam process by a novel high power ultrashort laser systems^23^ is not straightforward due to the very low close-to-ablation-threshold power required for LIPSS production^21^. Beam defocusing or using lenses with long focal lengths is a possible solution. For example, Mezera^24^ used defocused laser beam to increase LIPSS production 16-folds reaching 5.3 cm^2^/min on silicon and Faas^25^ used 525 W 8 ps laser source with 340 mm long F-theta focal distance to produce LIPSS on stainless steel with throughput of 12.5 cm^2^/min.

To further increase throughputs for the efficient use of high-power laser sources new fabrication methods have to be applied. These include polygon scanners providing scanning speeds above 100 m/s and multi-beam approaches^25,26^. Remarkable results were achieved by Schille^27^ combining hybrid polygon scanning system with fast polygon axis, perpendicular galvo axis and beam splitting to 4 sub-beams reaching 1300 cm^2^/min for LIPSS production on 40 × 50 mm stainless steel sample.

An alternative to polygon scanning systems can be beamsplitting by diffractive optical element (DOE) coupled with standard galvo scanning systems^28–31^. However, a large number of beams in order of thousands can be problematic due to the large separation distance of standard DOEs (typically > 10 times diffraction limited spot diameter) which results in a large irradiated area thus limiting scanning options due to scan-angle dependent distortions and limited scan area. Moreover, the large separation angles of standard DOEs require collimation to enable effective scanning without clipping and thus complicated multi-component scanning system^32^.

These issues can be addressed with a novel type of diffractive laser induced texturing (DLITe) DOE which allow dense texturing close to 50% of irradiated area. DLITe DOE generates a shaped pattern of light that is extremely dense, similar to that generated by interference patterning and does not require any additional collimation optics. As such it can be implemented as single element into existing setups. Additionally, sub-beams can be much closer (spot centre separations equal to the diffraction limited spot diameter) which enables effective scanning with minimized distortions. DLITe DOE is working in a regime where the splitter period is approximately X0.65 the laser input beam size. This texturing concept was introduced in previous theoretical work by the authors^33^, and this work is an experimental validation of this concept.

Thermal processes play an important role in high average power pulsed laser processing^34–36^. When using high pulse repetition frequency, residual heat from previous laser pulses in the same place increases the surface temperature of the material, which is called heat accumulation. The heat accumulation may result in the formation of structural defects on the surface. Uncontrolled heat accumulation at a very high repetition rate and low scanning speed leads to the onset of melting, oxidation, a pileup of material or thermal cracks^37–39^.

Thermal processes and surface temperature in ultrashort pulsed laser ablation are usually studied by numerical modelling and microscopy of the final resulting structure^34,40–42^. Time resolved measurements of laser heating, melting, vaporization and oxidation were done for laser pulse durations from milliseconds to nanoseconds by measuring reflectivity, transmissivity, emission and electrical conductance^43–45^. But for ultrashort pulsed laser ablation, only recently an infrared radiometry measurement system was developed and applied for ultrashort pulse laser micromachining^46^. Ultrashort pulsed laser melting during ablation was also investigated by time resolved reflectivity^47^.

In this work, a straightforward method for increasing the efficiency and fabrication speed of LIPSS-based functional surfaces is introduced. The input high-power laser beam is splitted by DLITe DOE into a square matrix of 51 × 51 sub-beams for simultaneous fabrication and placed before galvanometric scanner for fast beam matrix displacement over the sample. Additionally, a unique fast infrared radiometry system was applied for time-resolved thermal radiation measurements showing a correlation between thermal radiation changes and LIPSS production, thus presenting a simple way for potential closed-loop control during rapid large-scale fabrication of LIPSS-based functional surfaces.

## Beamspliting with diffractive laser induced texturing (DLITe) elements

**Figure 1:**
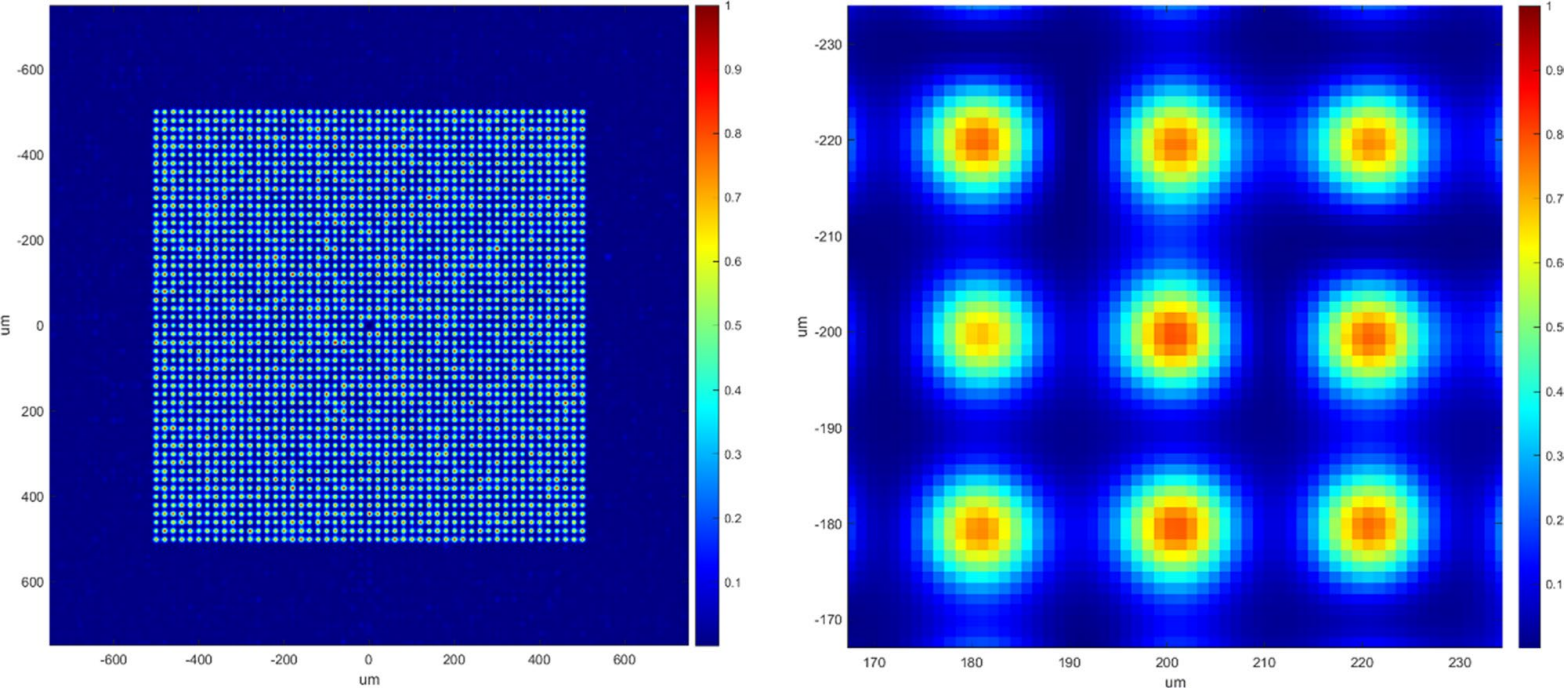
Simulation of the DOE splitter output at EFL = 100 mm, input beam 8 mm, wavelength 1030 nm (left: full field, right: zoom on 3 × 3 points). Slight Uniformity imperfections due to interference between neighbouring orders.

As shown in previous works^33^, a beam splitter DOE was designed to have a period equal to 0.65 times the input beam size, to achieve a dense pattern of intensity with a distance between maxima equal to the size of a diffraction limited spot. Input beam size was selected as 8 mm, to account for the optics clear aperture of 14 mm, resulting in a diffraction limited spot size of ~ 20 µm and a DOE period of 5150 µm which are typical values for tribology and hydrophobic applications^48–50^. To cover an area of 1 × 1 mm, a splitter with 51 × 51 was designed, having full angles of 10 × 10 mrad (separation angles 0.2 mrad × 0.2 mrad) and efficiency ~ 80%. To control zero order, a special 4 level design was employed causing the zero order to be defocused strongly at the work plane. In addition, the central order was designed as zero, as we expected some energy to be present there due to production tolerances. The simulation of the DOE splitter output is depicted in Fig. 1.

While all orders of the DOE are designed to have identical power (within standard deviation of 2%) mutual interference effects create a non-uniformity of the spots, calculated to have standard deviation of 28%. This results directly from the optimal ratio of beam size to DOE period for statistical area processing, defined in reference^33^ as 0.65.

## Results and discussion

**Figure 2:**
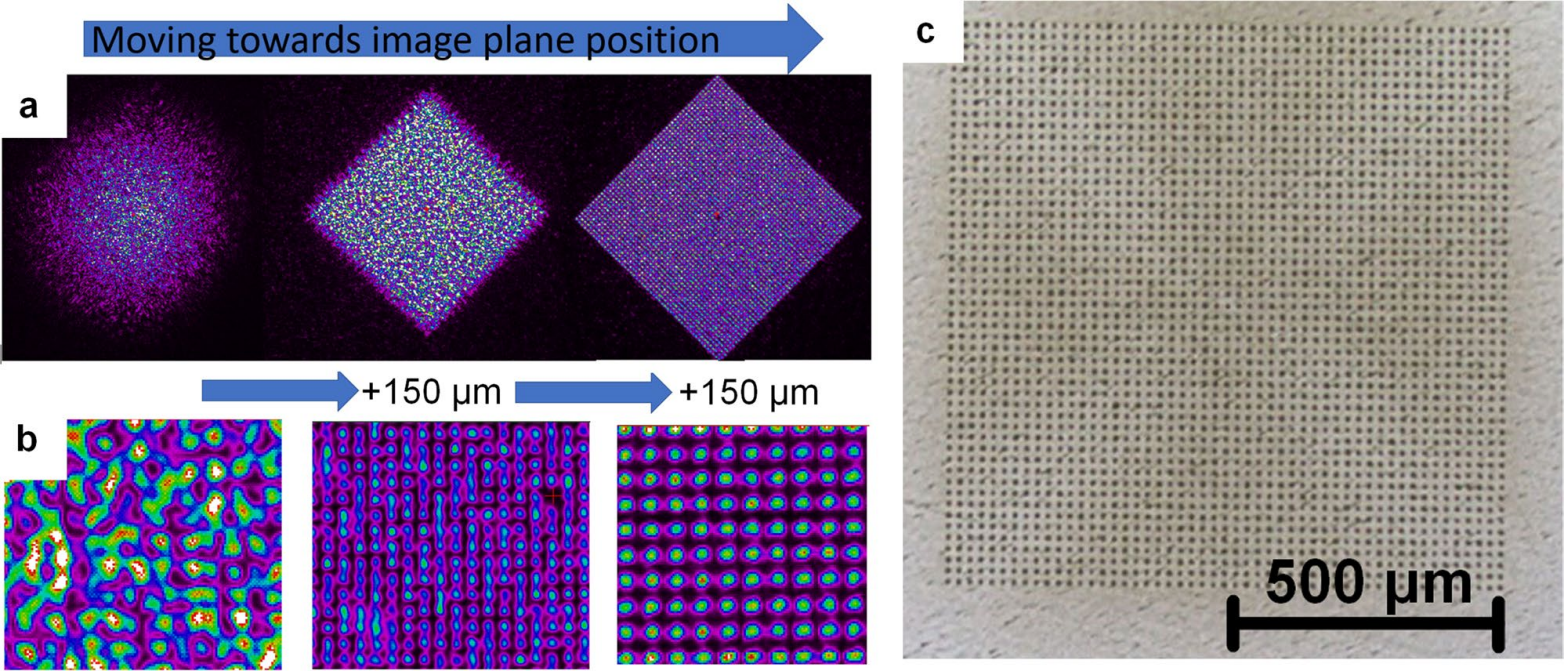
(**a**) coarse movement into image plane; (**b**) fine adjustment in image plane with a step of 150 µm in Z; (**c**) final pattern on stainless steel sample.

Square shaped matrix with 51 × 51 sub-beams can be attained only in the image plane of DLITe element (Fig. 2a) with a homogeneous sub-beam intensity in a range of ± 150 µm in z-axis (Fig. 2b). Thus, prior to sample patterning, a precise alignment was performed to reach homogenous intensity between 2601 sub-beams in square shaped matrix (Fig. 2c).

After the setup alignment, the pattern acquired in the optimal image plane shows a good correspondence with the theoretical simulation of the pattern in Fig. 1.

**Figure 3:**
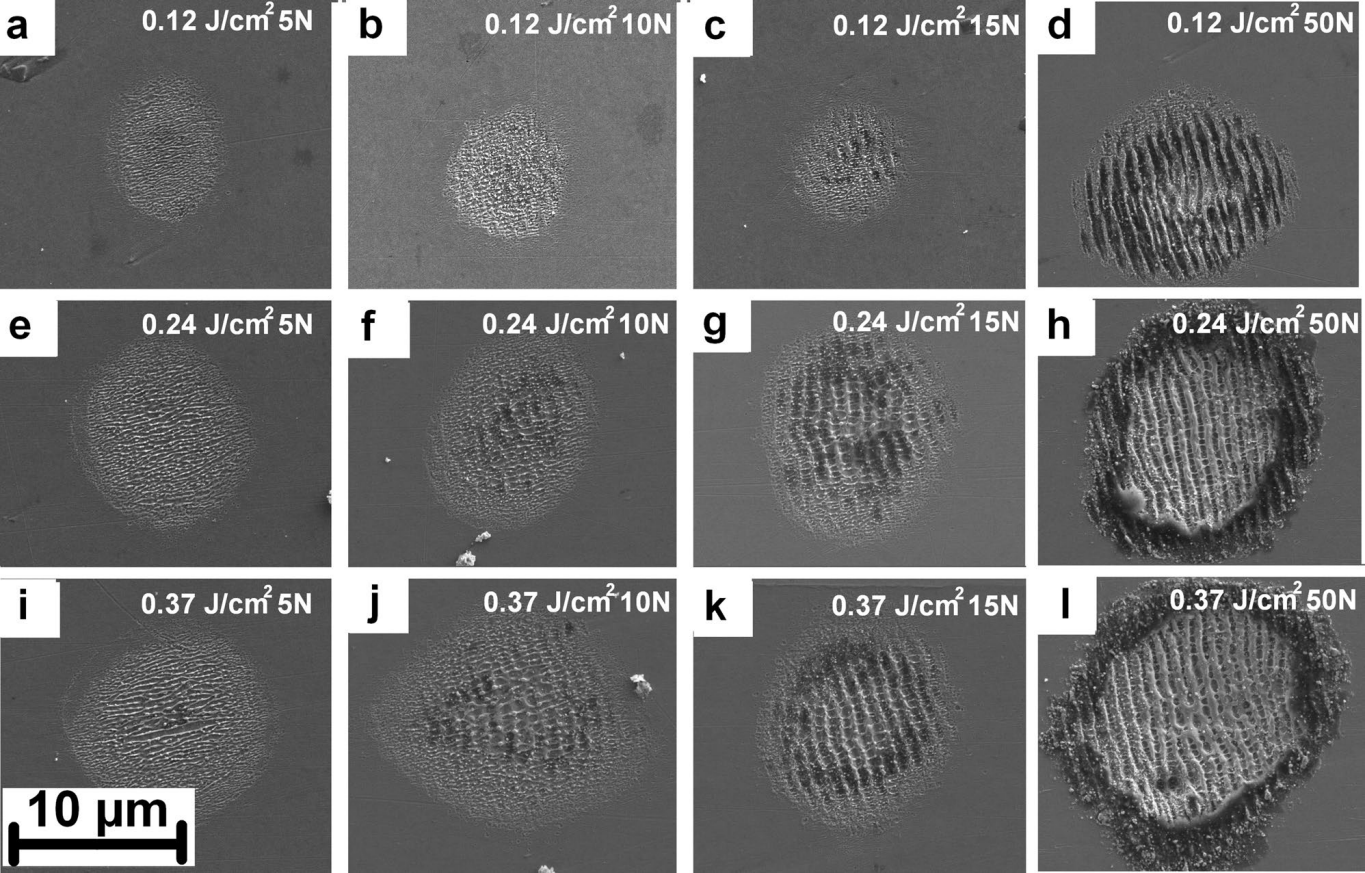
LIPSS evolution with the fluence and number of pulses (N).

In the following experiment, the LIPSS evolution with fluence and number of pulses (N) was studied. As depicted in Fig. 3, both LSFL and HSFL were fabricated at different fluence levels. These results indicate that the main factor affecting LIPSS fabrication is the amount of consecutive pulses.

Single pulse treatment did not result in LIPSS formation even for as high pulse energies as 4 mJ (0.49 J/cm^2^). By increasing the number of pulses to 5, HSFL with the periodicity of ~ 400 nm and parallel orientation to the beam polarization can be observed for all fluence levels between 0.12 – 0.37 J/cm^2^ (Fig. 3 a,e,i). However, as the fluence increases melting of HSFL is more pronouced decreasing the structure quality (Fig. 3 i). For the number of pulses between 10 and 15, LSFL with the periodicity of ~ 900 nm and perpendicular orientation to the beam polarization can be observed and starting to be more dominant for higher pulse counts (Fig. 3 d,h,l). LSFL are observed for a lower number of pulses when higher fluence is applied, as can be observed by coparing Fig. 3b and j. For pulse counts above 20 pulses, HSFL are visible only partially on top of LSFL and significantly melted (Fig. 3 h,l). The melted rim around the whole microcrater can be observed for the higest energy inputs resulting from high fluence and high number of pulses (Fig. 3 h,l).

**Figure 4:**
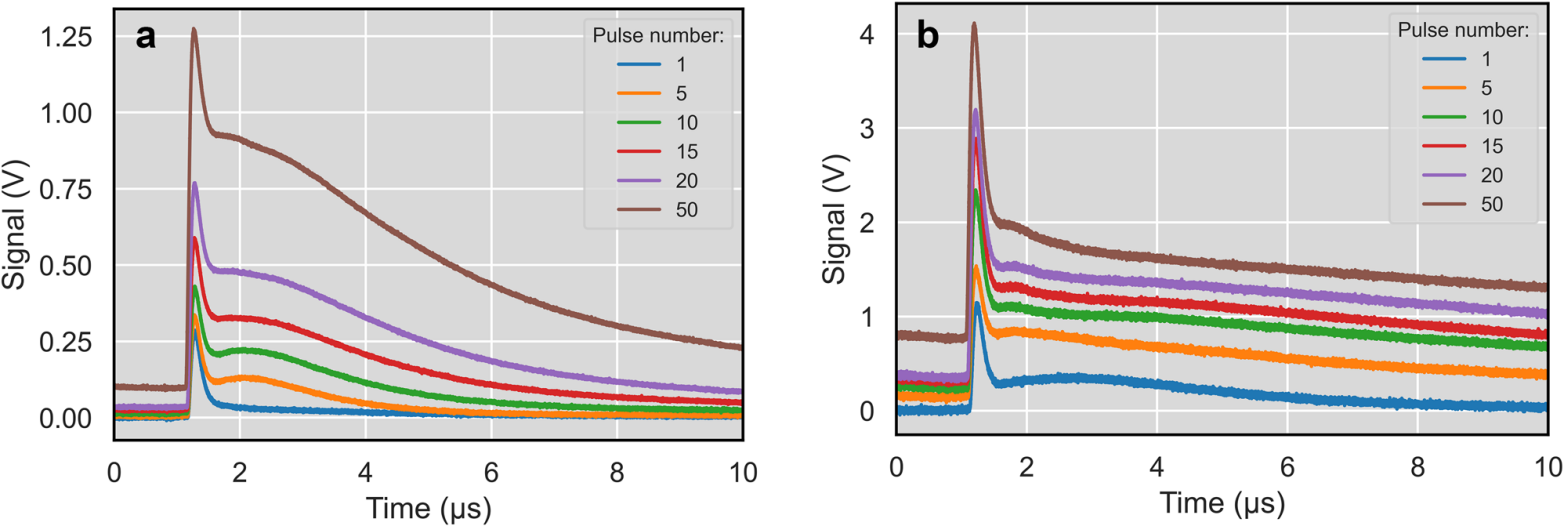
IR radiometry signal evolutions for different fluence and number of pulses: (**a**) 0.12 J/cm^2^, (**b**) 0.24 J/cm^2^.

**Figure 5:**
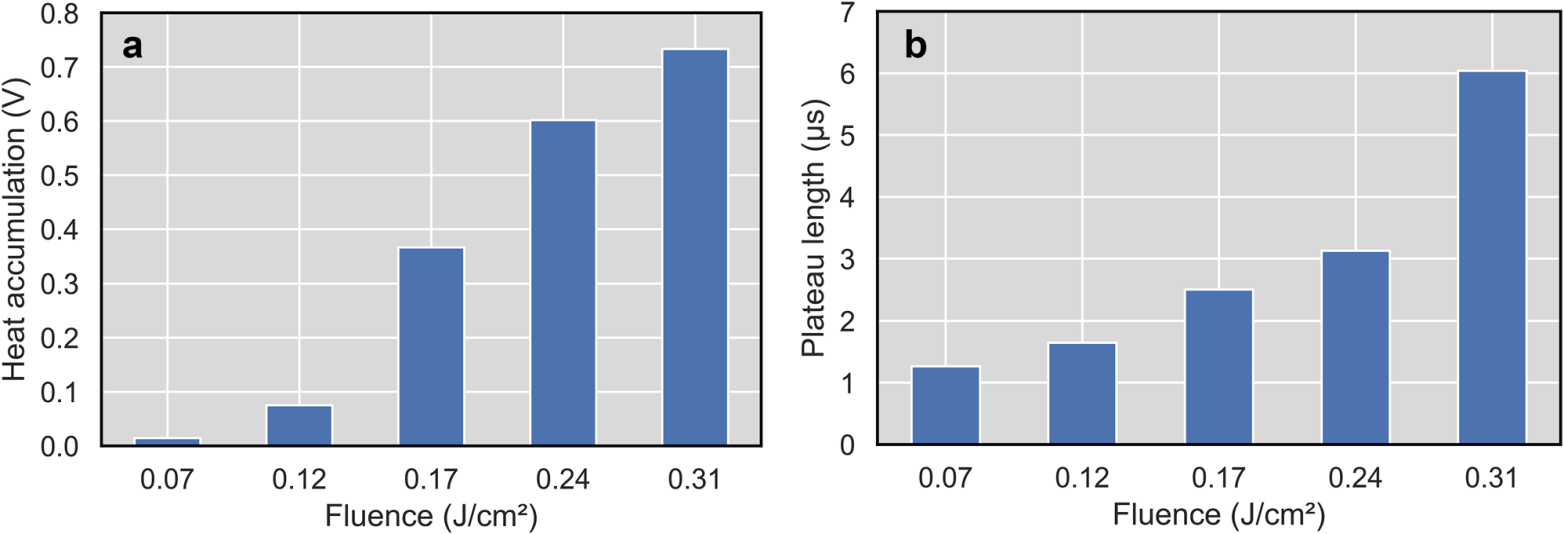
Average heat accumulation (**a**) and phase change plateau length (**b**) in dependance on laser fluence.

IR radiometry measurements are shown in Fig. 4. After a sharp peak the signal decreases to a certain value. In some cases, the first peak is followed by a smaller second peak. This evolution is very similar to phase change plateau^45^, where the phase change can be solidification or LIPSS formation. The plateau level significantly increases with the number of pulses. This can be caused by increasing of the melted area on the surface and thus radiating area (see Fig. 3). Heat accumulation signal is a signal before the next laser pulse – representing residual temperature from previous laser pulses^46^. This signal is also increasing with the number of pulses, mainly for the higher fluences (Fig. 4 b). The increase of heat accumulation with laser fluence is shown in Fig. 5a.

The phase change plateau also changes length with fluence and number of pulses. For higher fluence above 0.22 J/cm^2^ even two phase change plateaus appear for a higher number of pulses (> 5). It can be a sign of two different processes, e.g. LIPSS formation and solidification. The increase of plateau length can be explained by the bigger volume (depth) of melt on the surface. Averaged values from all number of pulses for each fluence are shown in Fig. 5b. The plateau length correlates well with the laser fluence. It means that it can be used for on-line process monitoring and control of LIPSS formation during the laser process. Also, heat accumulation signal can be used for on-line process control, because it increases mainly during the first pulses, where LIPSS are formed (1 to 15 pulses for higher fluences). According to its value the pulsing can be stopped on each place.

**Figure 6:**
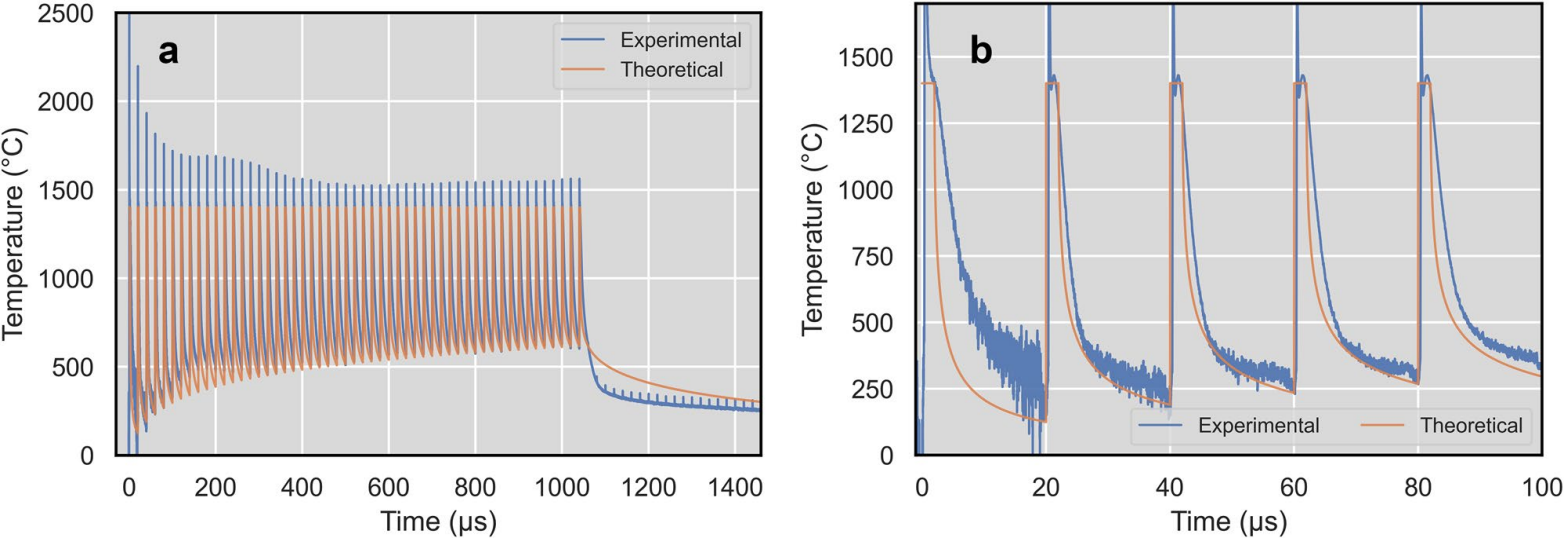
Temperature values during the multi-beam laser LIPSS formation for laser fluence of 0.12 J/cm^2^: (**a**) full temperature–time history of LIPSS formation (blue line experimental measurements, red line—theoretical approximation), (**b**) zoom of temperature–time history for the first five laser pulses.

An example of the resulting measured and calculated temperature evolutions is shown in Fig. 6. The measured temperatures are obtained from measured voltage signal by calibration based on observed solidification plateaus separately for each laser pulse. Such a dynamic calibration is a completely new approach and can have also some drawbacks. For example, the peak temperature for the first pulse rises up to 3500 °C, which may not be correct when taking into account the peak temperature of other pulses and the detector relatively slow response time compared to the laser pulse duration (60 ns compared to 2 ps). But this approach can reveal significant new insights into the problematics of the LIPSS formation. For example, in the case of 0.12 J/cm^2^ the phase change was observed already during the first pulse, although LIPSS was not observed on the SEM.

The presented model of heat accumulation has not included emissivity and absorptivity changes during LIPSS formation. Every laser pulse interaction is described as a heat source with constant temperature ${T}_{plateau}$ during LIPSS formation period (more details in the methods section) and it has not included nor chemical nor structure changes of laser irradiated material. Such simplification can be responsible for the difference between calculated and measured temperature–time history. The character of experimentally detected temperature decrease after the plateau is changing from pulse to pulse, especially in the beginning of the laser surface processing (Fig. 6b). The theoretical model has repeatable law of temperature–time history changes from pulse to pulse because it has a fixed geometrical coefficient $n$ and time interval of LIPSS formation ${t}_{plateau}$. The difference between the theoretical model and experimental data can help to discover the thermodynamics of multi-pulse LIPPS structure formation.

**Figure 7:**
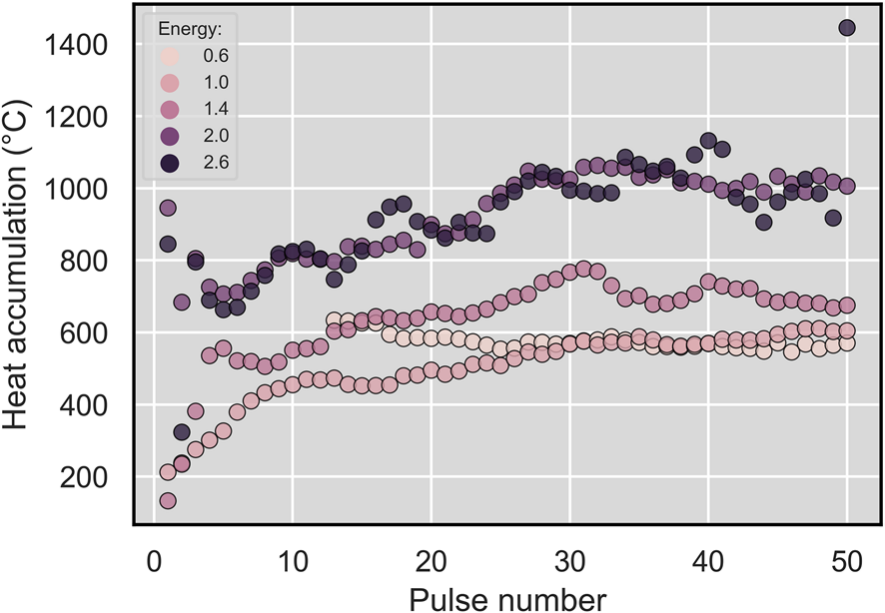
Heat accumulation temperatures for diferent fluences and laser pulses determined from the measurement and calibration.

The heat accumulation temperature shown in Fig. 6 increased fast during the first pulses over 400 °C and stabilizes around 600 °C at the end of the process. The detail values of heat accumulation temperature for all laser pulses and fluences are shown in Fig. 7. There can be found two groups of heat accumulation temperatures. First has the stabilized value around 600 °C (fluences 0.07 and 0.12 J/cm^2^) and the second around 1000 °C (fluences 0.24 and 0.31 J/cm^2^). The fluence 0.17 J/cm^2^ is somewhere in between them. This may be explained as two different process regimes with a transition between them. However, the temperatures around 600 °C for the laser fluence of 0.07 J/cm^2^ were unexpected due to the very low heat accumulation signal compared to the processes with fluence 0.12 and 0.17 J/cm^2^ (8 and 35 times lower). In this case, the explanation may be in the very localized areas of melting or LIPSS formation for the fluence of 0.07 J/cm^2^. In addition, a threshold to attain a stable value of the process was observed, which might be connected to the beginning of LIPSS formation. For low fluences, it takes a certain number of pulses to attain it (13 and 8 for 0.07 and 0.12 J/cm^2^). On the other hand for high fluences, it is reached already after the first pulse together with some oscillation during the first three pulses. Also, the heat accumulation temperature for the fluence of 0.31 J/cm^2^ is more unstable than in the case of 0.24 J/cm^2^, although in average showing the same results.

To cover larger areas, DLITe DOE was placed before the galvanometric scanner to stitch dot matrices. The advantageous square shape of the beam matrix allows 0% overlap and thus fast beam movement over the sample. To demonstrate the throughput, the sample area of 40 × 40 mm was covered by LSFL and HSFL with parameters corresponding to Fig. 3a and d, respectively. During this experiment, the scanning speed was set to 9 m/s and the laser system was operated at 100 kHz delivering 200 W of average power. The required time for patterning of the whole area reached 503 ms and 1205 ms thus reaching 1910 cm^2^/min and 797 cm^2^/min for HSFL and LSFL respectively. The real-time video of the process can be found in the supplementary material (Supplementary video S1) and a detail of processed surface in Supplemetary Fig. 1.

## Conclusion

The unique combination of the high-quality, high-energy laser beam with M2 < 1.2 and DLITe DOE element shaped the beam into the homogeneous square-shaped matrix of 51 × 51 sub-beams with a separation distance of 20 µm. Thus, resulting in a simultaneous microstructuring in 1mm^2^ area, 2601 times faster compared to the single beam approach. By adjusting the fluence and number of pulses additional nanostructures can be observed inside microholes produced by DLITe element. Both, LSFL and HSFL can be produced. LSFL were observed for pulse energies between 1 to 3 mJ (fluence of 0.12 to 0.37 J/cm^2^) and the number of pulses above 15 for 0,12 J/cm^2^ and above 10 for higher fluences. HSFL were observed for all tested fluences and the number of pulses between 5 to 10. For a higher number of pulses, LSFL start to dominate especially for high fluences. In addition, these results show a correlation with fast infrared radiometry measurements. Solidification plateaus were observed after each laser pulse, confirming that LIPSS formation is connected with the melting and solidification of the material. A new approach of using a derivation of the signal was applied to automatically calculate plateau length. Hence phase change plateau length and heat accumulation signal can be used for real-time quality control during LIPSS formation and solidification and for controlling the melt volume on the surface. Observation of solidification plateaus after each laser pulse enabled dynamic calibration of the measurement for obtaining temperatures. The resulting temperatures were compared with a theoretical model of heat accumulation. Heat accumulation temperatures from 200 to 1000 °C were observed from both the measurement and the model. The model can help to increase understanding of thermodynamics of multi-pulse LIPPS structure formation when comparing it with measurement for different laser pulse numbers. The temperature measurements revealed interesting changes in the physics of the laser ablation process, mainly two levels of heat accumulation and correlation of the heat accumulation fast increase to a stable value depicting the start of LIPSS formation.

DOE placed before a standard galvo scanner enables easy integration and stitching of the multi-beam pattern, demonstrating the potential for high-speed micro/nanostructuring over larger areas. By using the parameters for LSFL and HSFL production throughputs of 797 cm^2^/min and 1910 cm^2^/min were achieved on 40 × 40 mm area, respectively. Which is, to the best of our knowledge, the highest demonstrated throughput of LIPSS nanostructuring. Thus, opening new unprecedented opportunities towards a simple and robust rapid large-scale micro and nanostructuring with real-time quality control possibilities.

## Materials and methods

Stainless steel 304L samples with dimensions of 50 mm × 50 mm × 5 mm were mirror polished (Ra ~ 0.05 µm) and treated by Ytterbium based diode pumped solid state laser system Perla (HiLASE Centre, Czech Republic) emitting ultrashort pulses with pulse duration of 1.2 ps and M^2^ of 1.2 at 1030 nm and power up to 200 W. Repetition rate of 100 kHz and 50 kHz were used for the purpose of this experiment resulting in a pulse energy up to 4 mJ. The generated beam was guided through DLITe element MS-805-I-Y-A for beamsplitting into 51 × 51 matrix (Holo/Or Ltd., Israel), then into a high dynamic galvo-scanner system (Scanlab GmbH, Puchheim, Germany) and focused on a sample using telecentric F-theta lens with focal length of 100 mm.

The shape of the diffraction pattern and homogeneity of sub-beams were analysed by Basler ace acA3080-10um camera with a pixel size of 1.67 µm. The surface morphology was investigated by laser scanning confocal microscope, Olympus OLS5000 and scanning electron microscope, Tescan FERA 3 at electron energy of 5 kV.

Infrared (IR) radiometry system was composed of fast IR detector HgCdTe (Fermionics PV-11–1) cooled by liquid nitrogen, two off-axis paraboloid mirrors and a germanium filter. The wavelength range of the detector sensitivity was from 2 to 12 µm and its response time was 60 ns^46^.

IR radiometry measurements have shown in many cases a plateau of almost stable signal after a sharp peak. The plateau length was analyzed automatically by derivation of the IR signal curve. Before and after derivation also smoothing was applied. The time between maximum and minimum derivative is taken as the length of the phase change plateau. To obtain temperatures from the measured voltage values calibration was done based on the identified plateaus in the signal. First, a theoretical calibration table was created relating temperature and the response of the detector in voltage. The theoretical calibration table was calculated by integral of Planck’s law weighted by spectral sensitivity of the detector^46^. The calibration table was then adjusted so that the zero level matches the room temperature – the voltage column was subtracted by voltage value at room temperature.

The calibration was performed on the basis of the known solidification temperature of the material (1427°C^51^). The average signal in the top part of the plateau was used as the signal value of the solidification for the calibration. A multiplicative calibration coefficient was calculated for each plateau in the signal (each laser pulse) by dividing the value at the top of the plateau by the value from the calibration table corresponding to the phase transition temperature in volts. This coefficient accounts for the emissivity of the material and geometrical view factor of the optical focusing system^52^ and also for the size of the radiation area (comparable to the melted area). The obtained calibration coefficient was then used to multiply the voltage values from the calibration table. In this way, each plateau generated its own calibration coefficient and each part of the signal corresponding to one laser pulse (one period between pulses) has its own calibration.

Once all the calibration coefficients were found, the signal values were converted to temperature according to the corrected calibration table for every single peak. At the end of the process, the experiment signal was fully converted to the temperature, where all plateaus were aligned to the phase transition temperature of the material used.

### Theoretical calculations

**Figure 8:**
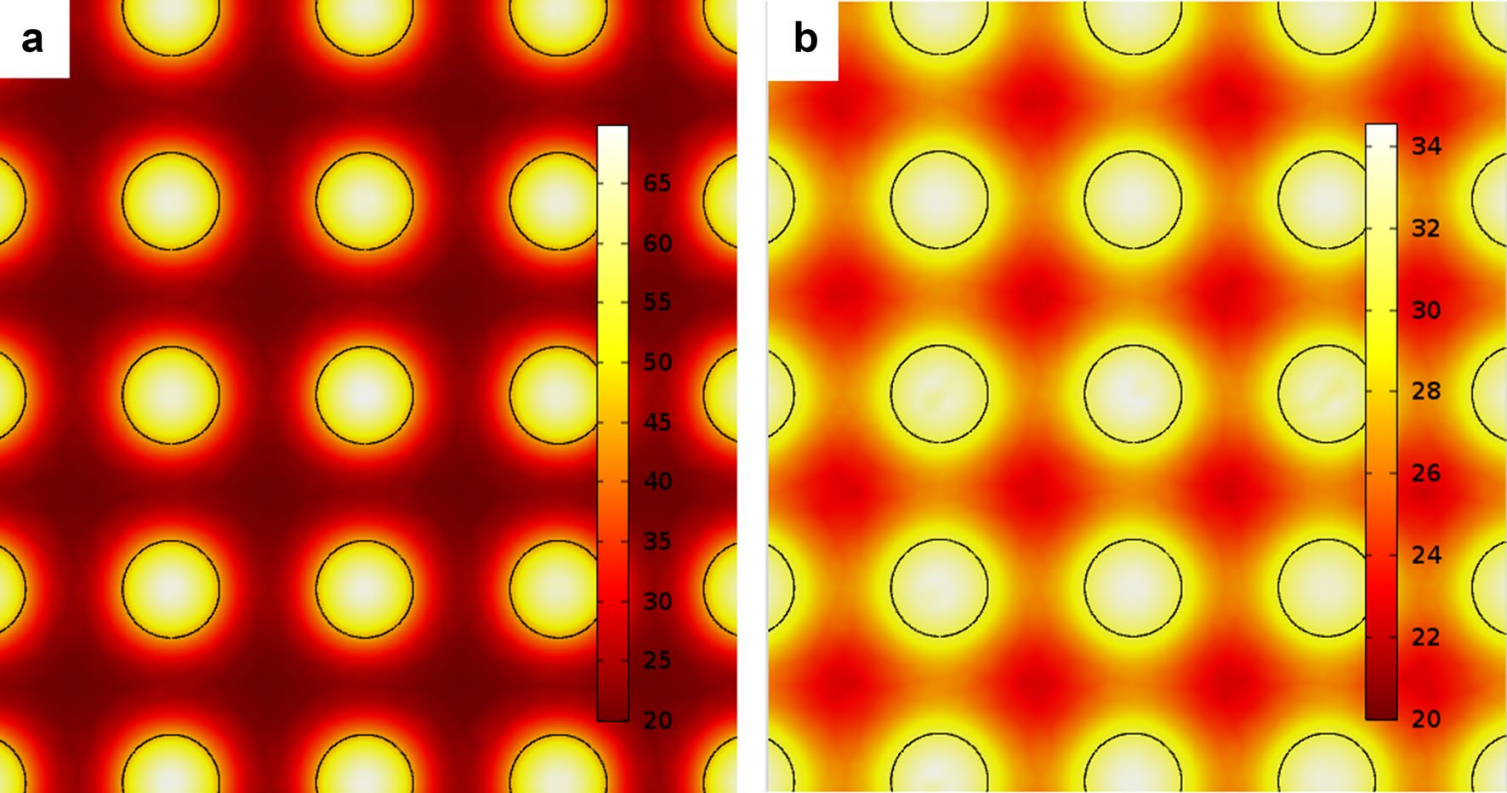
Temperature distribution after instantaneous heating of stainless steel surface with laser multi-spots up to the melting point: (**a**) at 1 µs after laser pulse, (**b**) at 3 µs after laser pulse.

The measured temperature evolutions during the experiment were compared to theoretical calculations defined as described here. The temperature evolution under laser irradiated surface is in direct dependence on intensity distribution on the material surface. For the case of planar, linear or point instantaneous heat source the temperature changes can be described as a solution of differential equation of conduction heat^37,53,54^

1 $$\Delta T=\frac{{Q}_{n}}{\sqrt{{(\pi \cdot \alpha \cdot t)}^{n}}}\cdot {e}^{-\frac{{r}_{n}^{2}}{t\cdot 4\cdot \alpha }}$$

where $\Delta T$—temperature changes under surface, ${Q}_{n}$—residual heat (which can be a function of heat source geometry), $n$—is a geometrical coefficient (for planar source $n=1$, for linear source $n=2$ and for point source $n=3$), $\alpha$—thermal diffusivity, ${r}_{n}^{2}$—distance from heat source (for the central point on surface ${r}_{n}^{2}=0$) and $t$—is the time period after the heat source is applied. In the case of multi-spot laser distribution on the irradiated surface, the heat source acts as an array of isolated laser spots at the very start ($t<1 \mathrm{\mu s}$) and later it becomes more similar to the planar source model (Fig. 8). From this point of view the temperature distribution under multi-spot laser irradiation can be defined by approximation of geometrical coefficient in the interval $1<n<3$ and it depends on laser spots diameter, their quantity and distance between them.

Full period of LIPSS formation includes the whole interval after the laser pulse irradiation till the moment of the plateau end ${t}_{plateau}=1-5\upmu s$ (Fig. 4). The temperature of plateau formation can be taken as a constant value equal to the melting point. The process of LIPSS formation during plateau can be described as an uninterrupted heat source and Eq. 1 will be represented as an integral^54^:

2 $${T}_{plateau} =\frac{1}{\sqrt{{(\pi \cdot \alpha )}^{n}}}{\int }_{0}^{{t}_{plateau}}\frac{{Q}_{n}(t)}{{t}^{n/2}}dt$$

where ${T}_{plateau}$—temperature during plateau and ${Q}_{n}(t)$ is the quantity of heat, which is transferred from the laser spot to the material surface during LIPSS formation. The temperature of LIPSS formation can be taken equal to the melting point. During LIPSS formation the transferred heat ${Q}_{n}(t)$ is decreasing due to decreasing of temperature gradient under laser spots. The temperature of LIPSS formation and time during plateau formation can be expressed in simple form:

3 $$q(t)={T}_{plateau}\cdot {t}^{n/2}$$

where $q(t)$ is a term that includes thermal characteristics of material and strength of heat source in LIPSS formation area. Term values $q(t)$ can be defined numerically in whole plateau duration and then it will be used for temperature–time history evolution after every laser pulse:

4 $$\Delta T={\sum }_{0}^{m}{\sum }_{0}^{k}{q}_{mk}/{t}_{mk}^{n/2}$$

where $m$—is number of applied laser pulse, $k$—is number of evaluated therm in the LIPPS formation plateau, ${t}_{mk}$—time interval between evaluated point $mk$ and actual temperature–time history point. For the applied multi-beam process with 10 µm diameter spots and 20 µm distance between them, the geometrical coefficient was defined es equal to $n=1.5$. The input parameter for this heat accumulation model is the full plateau duration, which is needed for the determination of terms ${q}_{mk}$. For laser beam energy of 1 mJ, it was taken ${t}_{plateau}=2 \mu s$.

## Supplementary Information

Supplementary Information 1.

Supplementary Video 1.

Supplementary Information 2.
