# Supplementary material for: LIPSS-based functional surfaces produced by multi-beam nanostructuring with 2601 beams and real-time thermal processes measurement
Source: Sci Rep. 2021 Nov 25;11:22944. doi: 10.1038/s41598-021-02290-3 (PMC8617047; doi:10.1038/s41598-021-02290-3)
Supplement: Supplementary file 3 — Supplementary Information 2. [file 41598_2021_2290_MOESM3_ESM.docx]

Supplementary video S1. Real-time process video of a sample covered by HSLF with a productivity of 1910 cm^2^/min.
